# Morphine acts in vitro to directly prime nociceptors

**DOI:** 10.1177/17448069241260348

**Published:** 2024-06-03

**Authors:** Eugen V. Khomula, Jon D. Levine

**Affiliations:** 1 Department of Oral & Maxillofacial Surgery, 8785 University of California at San Francisco, San Francisco, CA, USA; 2 Department of Medicine, Division of Neuroscience, and UCSF Pain and Addiction Research Center, 8785 University of California at San Francisco, San Francisco, CA, USA

**Keywords:** Morphine, opioid-induced hyperalgesic priming, nociceptor, prostaglandin E_2_, sensitization, patch-clamp electrophysiology, rheobase

## Abstract

Hyperalgesic priming is a preclinical model of the transition from acute to chronic pain characterized by a leftward shift in the dose-response curve for and marked prolongation of prostaglandin E_2_ (PGE_2_)-induced mechanical hyperalgesia, in vivo. In vitro, priming in nociceptors is characterized by a leftward shift in the concentration dependence for PGE_2_-induced nociceptor sensitization. In the present in vitro study we tested the hypothesis that a mu-opioid receptor (MOR) agonist opioid analgesic, morphine, can produce priming by its direct action on nociceptors. We report that treatment of nociceptors with morphine, in vitro, produces a leftward shift in the concentration dependence for PGE_2_-induced nociceptor sensitization. Our findings support the suggestion that opioids act directly on nociceptors to induce priming.

## Introduction

While opioid analgesics remain amongst the most effective treatments for moderate-to-severe pain,^1–6^ their clinical use is limited by serious adverse effects including, analgesic tolerance, addiction, opioid-induced hyperalgesia (OIH), and pain chronification.^7–11^ We have established a preclinical model of the transition from acute to chronic pain, referred to as hyperalgesic priming, a form of long-lasting nociceptor neuroplasticity manifested by a left shift in dose-dependence and marked prolongation of mechanical hyperalgesia in response to pronociceptive mediators, prototypically prostaglandin E_2_ (PGE_2_), and shown that two commonly used clinical opioids, fentanyl and morphine, produce both OIH and opioid-induced hyperalgesic priming (OIHP).^12–25^ More recently we have shown that small-diameter dorsal root ganglion (DRG) neurons, nociceptors, cultured from rats that had been primed by fentanyl, in vivo, express strong sensitization by subsequent in vitro administration of fentanyl (in vitro equivalent of OIH), and enhanced sensitization by in vitro administration of a low concentration of PGE_2_ (in vitro equivalent of hyperalgesic priming). These results support the hypothesis that nociceptor neuroplasticity induced by in vivo administration of opioid analgesics persist in DRG neurons cultured from primed rats, providing an in vitro model for studying nociceptor mechanisms involved in *expression* and *maintenance* of OIH and OIHP.^24,25^ In the present experiments we determined if hyperalgesic priming (OIHP) could be induced in vitro by brief morphine exposure of DRG neurons cultured from opioid naïve animals, extending our existing model of hyperalgesic priming to a “full in vitro” model, priming in a dish, where nociceptor mechanisms of *induction*, *expression* and *maintenance* of OIHP could be elucidated at the cellular level.

## Materials and methods

### Culturing rat DRG neurons

Primary DRG cultures were prepared from 220 to 235 g adult male Sprague-Dawley rats (Charles River Laboratories, Hollister, CA, USA), as described previously.^20,24–26^ All experimental protocols were approved by the Institutional Animal Care and Use Committee at the University of California, San Francisco, and adhered to the National Institutes of Health *Guide for the care and use of laboratory animals*. Until used for culture preparation, animals were housed three per cage, under a 12-h light/dark cycle, in a temperature- and humidity-controlled animal care facility at the University of California, San Francisco. Food and water were available in home cages, ad libitum. Effort was made to minimize number of animals used and their suffering. Under isoflurane anesthesia, rats were decapitated, and the dorsum of their vertebral column surgically removed; L_4_ and L_5_ DRGs were then rapidly extirpated, bilaterally, chilled on ice and desheathed in Hanks’ balanced salt solution (HBSS). DRG were treated with 0.25% collagenase Type 4 (Worthington Biochemical Corporation, Lakewood, NJ, USA) in HBSS, for 18 min at 37°C, and then with 0.25% trypsin (Worthington Biochemical Corporation) in calcium- and magnesium-free PBS (Invitrogen Life Technologies, Grand Island, NY USA) for 6 min, followed by three washes and trituration in Neurobasal-A medium (Invitrogen Life Technologies) to produce a single-cell suspension. This suspension was centrifuged at 1000 RPM for 3 min followed by re-suspension in Neurobasal-A medium supplemented with 50 ng/mL nerve growth factor, 100 U/mL penicillin/streptomycin, B-27, GlutaMAX and 10% FBS (Invitrogen Life Technologies). Dissociated DRG neurons were plated on cover slips and incubated at 37°C in 3.5% CO_2_ for at least 24 h before they were used in electrophysiology experiments. One hour after plating, 1 mL of culture media was gently added. The next day plated coverslips were fractured to obtain 6-8 individual pieces for use in electrophysiology experiments, and another 1 mL of culture media was added. Cultured neurons were used within 24-72 h after preparation.

### Whole-cell patch-clamp electrophysiology

Following placement of a coverslip fragment plated with cells in the recording chamber, culture medium was replaced with the solution used to perform electrophysiology; Tyrode's solution containing 140 mM NaCl, 4 mM KCl, 2 mM MgCl_2_, 2 mM CaCl_2_, 10 mM glucose, and 10 mM HEPES, adjusted to pH 7.4 with NaOH, with an osmolarity of 310 mOsm/kg. The recording chamber had a volume of 150 µl, and its perfusion system a flow rate of 0.5–1 ml/min. Electrophysiology experiments were conducted at room temperature (20–23°C).^25^

Cells to be recorded from were identified as neurons by their double birefringent plasma membrane.^27,28^ Whole-cell patch-clamp recordings, performed in current clamp mode, were used to evaluate for changes in neuronal excitability. Holding current was adjusted to maintain membrane potential at −70 mV. Rheobase, defined as the minimum magnitude of a current step needed to elicit an action potential (AP), was determined through a testing protocol utilizing sequential square wave pulses with current magnitude increasing by constant steps, until an AP was elicited. An initial estimate of rheobase was made with 500-pA increments in stimulus current (0.5 – 4 nA). The increments were then adjusted to achieve 5%–10% precision of the rheobase estimate.^25,26^

Recording electrodes were fashioned from borosilicate glass capillaries (0.84/1.5 mm i.d./o.d., Warner Instruments, Holliston, MA, USA) using a Flaming/Brown P-87 microelectrode puller (Sutter Instrument Co, Novato, CA, USA). After being filled with a solution containing 130 mM KCl, 10 mM HEPES, 10 mM EGTA, 1 mM CaCl_2_, 5 mM MgATP, and 1 mM Na-GTP; pH 7.2 (adjusted with Tris-base), resulting in 300 mOsmol/kg osmolarity, recording electrode resistance was approximately 2 MΩ. Junction potential was not adjusted. Measured at the end of recordings, without compensation, series resistance was below 10 MΩ. Recordings were conducted using an Axon MultiClamp 700 B amplifier, filtered at 20 kHz, and sampled at 50 kHz through an Axon Digidata 1550B controlled by pCLAMP 11 software (all from Molecular Devices LLC, San Jose, CA, USA).^25^ To ensure the stability of baseline current, drugs were applied at least 5 min after the establishment of whole-cell configuration.

### Drugs and media

The following drugs were used in this study: Morphine sulfate salt pentahydrate, prostaglandin E_2_ (PGE_2_), NaCl, KCl, MgCl_2_, CaCl_2_, NaOH, MgATP, Na-GTP, D-Glucose, 4-(2-Hydroxyethyl)piperazine-1-ethanesulfonic acid (HEPES) and Ethylene glycol-bis(2-aminoethylether)-N,N,N′,N′-tetraacetic acid (EGTA) (Sigma Aldrich, St Louis, MO, USA), Collagenase Type 4, Trypsin (Worthington Biochemical Corporation, Lakewood, NJ, USA), Calcium- and Magnesium-free HBSS, Calcium- and Magnesium-free PBS, Neurobasal-A medium, B-27 supplement, GlutaMAX, Fetal Bovine Serum (FBS), and rat (recombinant) nerve growth factor (NGF)-beta (Invitrogen Life Technologies, Grand Island, NY USA).

The stock solution of morphine sulfate (1 mM) was prepared freshly from powder (0.76 mg/mL, in purified water) on the day of the experiment and stored before use at 4°C. The final concentration of morphine in perfusion solution was 20 µM (1:49 dilution of the stock solution in Tyrode’s solution).^29,30^

The stock solution of PGE_2_ was prepared in absolute ethanol (1 mg/mL, 2.83 mM) and stored as tightly sealed 10 µL aliquots at −80°C. For in vitro electrophysiology, a final stock solution of PGE_2_ was prepared in distilled water (25 µM, 1:112 dilution) on the day of the experiment and stored before use at 4°C. Concentrations of PGE_2_ in the perfusion solution were 10 and 100 nM, as used previously to detect priming in vitro^25^; 100 nM was produced by a 1:249 dilution of the secondary stock solution, in Tyrode’s solution, performed just before it was used in experiments, and similarly 10 nM was produced by a 1:9 dilution of 100 nM solution.

### Statistical analysis

Rheobase was measured before and again 5 min after application of PGE_2_. Magnitude of the sensitizing effect of PGE_2_ was expressed as percentage reduction in rheobase (i.e., its value before PGE_2_ administration [baseline] was subtracted from its value after, then the difference was divided by baseline). Only neurons sensitized above baseline by at least 10%, by either the lower (10 nM) or higher (100 nM) concentration of PGE_2_, applied to the same neuron 10 min apart, were considered PGE_2_ responsive and included in our analysis.^25,26^ Two-way ANOVA followed by Sidak's multiple comparisons test was used to analyze the effect of morphine on reduction in rheobase induced by PGE_2_ (10 and 100 nM).

Prism 10.2 (GraphPad Software) was used to generate graphics and perform statistical analyses; *p < .05* is considered statistically significant. Data are presented as mean ± SEM.

## Results

In vivo*,* hyperalgesic priming is usually detected by the presence of a marked prolongation of PGE_2_-induced hyperalgesia, still unattenuated after 4 h^15,17,20,22,31^; in control rats the duration of PGE_2_ hyperalgesia is ∼ 1 h.^12^ Since it is not practical to record from patch-clamped nociceptors for 4 h, to reveal priming in vitro, we have relied on another in vivo feature of hyperalgesic priming, a leftward shift in the dose dependence of PGE_2_-induced hyperalgesia.^14^ Thus, it is possible to detect primed neurons in vitro by their enhanced response to a low concentration of PGE_2_.^25^ However, since only ∼60% of cultured small-diameter DRG neurons respond to high concentrations of PGE_2_,^32^ it is not possible to distinguish between PGE_2_-responsive and nonresponsive neurons by their response to a low concentration of PGE_2_. Therefore, response to a second, higher concentration of PGE_2_, which sensitizes control nociceptors, is required to establish that a nociceptor is PGE_2_ responsive. Previously, we used two concentrations of PGE_2_, 10 nM and 100 nM, to demonstrate that hyperalgesic priming induced by fentanyl, in vivo, is associated with a significantly increased sensitizing effect of PGE_2_, in vitro, a leftward shift of the concentration-response curve in nociceptors from primed animals.^25^ That is, nociceptors cultured from in vivo primed rats remain primed when tested in vitro. In the present experiments we have used the same approach, to examine the hypothesis that a relatively short (1 h) exposure of cultured DRG neurons to morphine, in vitro*,* produces a leftward shift for sensitization induced in PGE_2_-responsive nociceptors, studied 24-72 h following exposure to morphine, constituting opioid-induced hyperalgesic priming (OIHP). These findings demonstrate, in vitro, that opioids act directly on nociceptors to induce priming.

Rheobase, the minimal sustained current required to generate an action potential (AP), was selected as our measure of neuronal excitability as it is a well-defined electrophysiological property that reflects excitability in DRG neurons, including nociceptors.^24,25,33^ Percentage decrease in rheobase was used as our measure of nociceptor sensitization. The effect of PGE_2_ on rheobase was evaluated in putative C-type nociceptive (small-diameter) DRG neurons (soma diameter <30 µm).^32,34–36^

**Figure 1.**
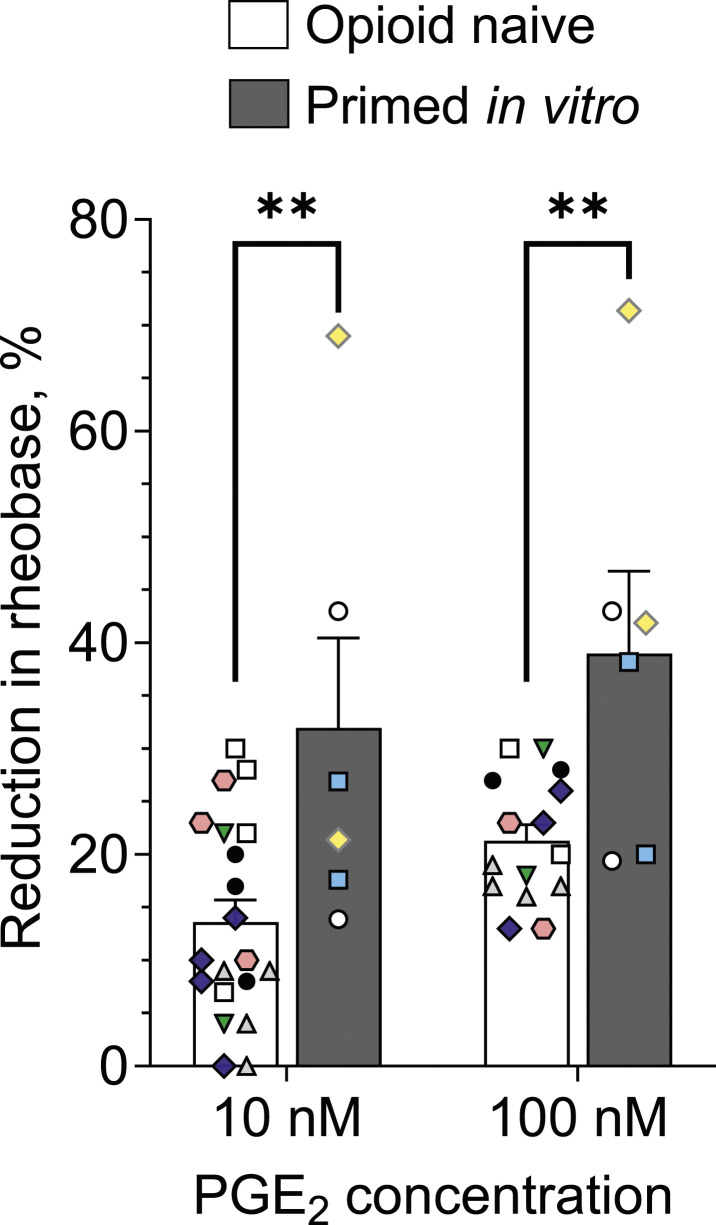
Prior in vitro exposure of small-diameter DRG neurons to morphine produces persistent enhancement of PGE_2_-induced nociceptor sensitization. DRG neurons were exposed to morphine (20 µM) in their culturing media, for 1 h in a CO_2_ incubator, 24 h after establishing cultures. On the following 3 days after morphine exposure (48-96 h after establishing cultures), patch-clamp electrophysiology recordings were performed on small-diameter (≤30 µM) DRG neurons from control (opioid naïve) and morphine-treated cultures (depicted by the white and dark grey bars, correspondingly). Bars show average magnitudes of reduction in rheobase, relative to pre-administration baseline (measured before the first application of PGE_2_), and 5 min after application of 10 nM and then 100 nM. 10 nM PGE_2_ was applied first, and then, 10 min later, its concentration was increased to 100 nM (cumulative concentration dependence). Symbols show individual values. Only those neurons with a reduction in rheobase not less than 10%, for either the higher or lower concentration of PGE_2_, were considered for analysis. In morphine-treated neurons the effect of both concentrations of PGE_2_ was significantly greater than their effects in controls (two-way ANOVA: effect of treatment, ****p < .0001, F_(1,43)_ = 20.2; Sidak's multiple comparisons test: t_(43)_ = 3.3, ** adjusted *p* = .004 for 10 nM; t_(43)_ = 3.1, ** adjusted *p* = .008 for 100 nM). Number of cells in control group: *n* = 20 for 10 nM and *n* = 15 for 100 nM; in primed group: *n* = 6 for both 10 nM and 100 nM. Number of rats (different culture preparations): six in control and three in primed group. Symbols with different color/shape combinations were used to show cells from different rats.

In the present experiment we compared the reduction in rheobase produced by 10 nM followed by 100 nM PGE_2_, in two groups of nociceptors, i.e. opioid naïve and morphine-treated (20 µM for 1 h), in vitro. In cultures of opioid naïve DRG neurons the magnitude of sensitization of small-diameter nociceptive neurons was 13.6 ± 2.1% (*n* = 20) for 10 nM PGE_2_ and 21.3 ± 1.5% (*n* = 15) for 100 nM PGE_2_. In nociceptors that were exposed to morphine (primed), in vitro, PGE_2_-induced reduction of rheobase was 32 ± 9% (*n* = 6) for 10 nM PGE_2_ and 39 ± 8% (*n* = 6) for 100 nM PGE_2_, which is significantly greater for both concentrations of PGE_2_ than in control neurons (Figure 1). The percentage of small-diameter neurons sensitized by 100 nM PGE_2_ was similar in control (64%, 20/31) and morphine-primed (67%, 6/9) nociceptors.

Thus, in vitro treatment with morphine enhances nociceptor sensitization by the subsequent exposure to PGE_2_. This finding provides support for our hypothesis that a direct action of opioids on nociceptors induces priming (opioid-induced hyperalgesic priming, OIHP). Altogether, our findings support the suggestion that opioids induce priming by a direct action on nociceptors, establishing a model for studying mechanisms underlying OIHP (including its induction, expression and maintenance), in vitro.

## Discussion

Mu-opioid receptor (MOR) agonist opioid analgesics can produce a hypersensitivity to noxious stimuli (OIH) and long-lasting pain exacerbation (pain chronification).^37–39^ We have recently shown that two important clinically used MOR agonist opioid analgesics, fentanyl^20,24^ and morphine,^17,22^ as well as two biased MOR-agonists, PZM21 and TRV130,^40^ can produce both OIH and opioid-induced hyperalgesic priming (OIHP). Since the action of MOR agonists on nociceptors plays an important role in their in vivo pronociceptive effects,^41–43^ increasing nociceptor excitability, we focused our attention on developing an in vitro model of OIHP using in vitro patch-clamp electrophysiology, to improve our understanding of the cellular mechanisms in the nociceptor mediating OIHP.

We have recently demonstrated that nociceptor neuroplasticity induced by in vivo administration of fentanyl persists in DRG neurons cultured from primed rats, resembling properties of OIH (enhanced sensitization by fentanyl) and OIHP (enhanced sensitization by PGE_2_).^24,25^

Consistent with our previous in vivo findings,^17,22^ in the present experiments we observed enhanced PGE_2_-induced sensitization (reduction of rheobase) in small-diameter DRG neurons treated with morphine (*primed*) in vitro, by both the lower (10 nM) and higher (100 nM) concentrations of PGE_2_, confirming our hypothesis that OIHP-like neuroplasticity can be *induced* in vitro. Of note, the magnitude of the enhancement was similar to that observed in vitro following in vivo fentanyl-induced OIHP.^25^ The enhancement of PGE_2_-induced nociceptor sensitization is not just a proportional increase in response to both lower and higher concentrations of PGE_2_ but demonstrates a leftward shift in the concentration dependence of PGE_2_-induced decrease in rheobase. Moreover, our finding that the percentage of PGE_2_-sensitive small-diameter neurons (those sensitized by 100 nM PGE_2_) was similar in control and morphine-primed nociceptors, and also to the percentage of sensitized nociceptors in previous studies of PGE_2_-induced nociceptor sensitization,^32^ supports the suggestion that priming is enhancing sensitivity to PGE_2_ in nociceptors that are PGE_2_-responsive rather than recruiting neurons previously unresponsive to PGE_2_. Our finding of priming-like neuroplasticity in cultured DRG neurons that have lost their terminals in peripheral tissues and spinal dorsal horn, during culture preparation, support the suggestion that priming in nociceptors can exist in the soma and, in this model, is induced by a direct action of morphine on MORs, which are expressed in the nociceptor’s soma.

While the present experiments have demonstrated in vitro that morphine induces priming-like neuroplasticity in nociceptors, and there is strong evidence for MOR signaling in nociceptors,^20^ it is noted that the current experimental design does not completely exclude the contribution of an indirect mechanism of induction of this neuroplasticity via action of morphine on non-neuronal DRG cells, which could produce signaling molecules and thus at least partially contribute to the induction of neuroplasticity in nociceptors. DRG cultures were prepared from whole DRGs not cleared of non-neuronal cells. While it has been reported that these cells express MOR and can act indirectly to contribute to sensitization of DRG neurons^44,45^ by an activated of immune response, *in vivo*^46–48^ However, we prepared low-density DRG neuronal cultures that were used within 3 days, at which time there is minimal growth of non-neuronal cells and nociceptor neurites. This approach has allowed us to patch nociceptors without neighboring non-neuronal cells within > 100 µm. Our calculations suggest that in the in vitro configuration used in the presented experiments a possible signaling between nociceptors and non-neuronal cells would be attenuated by roughly four orders of magnitude compared to in vivo adjacent location.

In the present study experiments were performed on nociceptors from male rats, to parallel our recent finding of enhanced in vitro nociceptor sensitization by PGE_2_ after priming in males^25^ to demonstrate induction of priming-like neuroplasticity, in vitro. Our previous studies, while demonstrating that both a selective MOR-agonist DAMGO^19^ and a clinically used opioid analgesic, fentanyl *(unpublished observation*), induce priming in both male and female rats, have revealed that the underlying mechanisms were different. Thus, the detailed dose response to PGE_2_, changes in electrophysiological properties other than rheobase, underlying cellular mechanisms, including ionic channels, and involved neuronal populations in nociceptors from both male and female animals are future avenues of study.

Three forms of OIHP have been reported. Type I is reversed by cordycepin,^17,20,44^ Type II by the combination of a Src and a MAPK inhibitor,^18,20^ and a third type, produced by biased MOR agonists, is independent of mechanisms mediating Type I and Type II priming.^40^ Both our in vivo and in vitro findings show that an analgesic dose of fentanyl induces priming sharing *maintenance* mechanisms with both Type I and Type II priming. Morphine, however, produced only Type I OIHP.^17,22^ While it is unlikely that nociceptor populations and type of OIHP induced by morphine in vitro is different from those observed in vivo, the direct answer to this question could be a future direction using our now established in vitro model.

In conclusion, our results indicate that morphine induces neuroplasticity in nociceptors, in vitro, resembling properties of OIHP (enhanced sensitization by PGE_2_ with a leftward shift in its concentration dependence), providing, for the first time, a “full in vitro” model of OIHP where nociceptor mechanisms of *induction*, *expression* and *maintenance* of OIHP could be directly elucidated.

## Data Availability

Research materials (including data) related to this paper will be available upon request. *
